# Anthocyanin bio-fortified colored wheat: Nutritional and functional characterization

**DOI:** 10.1371/journal.pone.0194367

**Published:** 2018-04-04

**Authors:** Saloni Sharma, Venkatesh Chunduri, Aman Kumar, Rohit Kumar, Pragyanshu Khare, Kanthi Kiran Kondepudi, Mahendra Bishnoi, Monika Garg

**Affiliations:** National Agri-Food Biotechnology Institute, Mohali, Punjab, India; Institute of Genetics and Developmental Biology Chinese Academy of Sciences, CHINA

## Abstract

Colored wheat, rich in anthocyanins, has created interest among the breeders and baking industry. This study was aimed at understanding the nutritional and product making potential of our advanced, high yielding and regionally adapted colored wheat lines. Our results indicated that our advanced colored wheat lines exhibited higher anthocyanin content and antioxidant activity than donor wheat lines and it varied in the order of white<purple<blue<black wheat. UPLC chromatogram revealed that anthocyanin composition and peak pattern is not only dependent on donor genotype but also background of recipient genotype. Interestingly, the purple wheat extract showed highest anti-inflammatory effect and followed the trend of white<blue<black<purple. Nutritional (carbohydrates, sugar, protein, ash, dietary fibre and vitamins) and processing parameters in relation to end-use quality (SDS sedimentation, gluten content, alveograph) of advanced colored lines were similar to high yielding white wheat cultivar. Colored wheat lines showed high iron and zinc content compared to white wheat indicating double bio-fortification. Therefore, our advanced colored wheat lines with high anthocyanin, iron and zinc contents showed antioxidant and anti-inflammatory activity and possessed desirable features for product making and commercial utilization.

## Introduction

Cereals take an important place among all the food items and play key role for a healthy diet. Among the cereals, wheat is the leading one, devoted to multiple uses such as bread, noodles and biscuits. Wheat is a good source of starch, proteins, minerals and dietary fibre and is major contributor towards daily caloric requirements of wheat consuming population. Further enhancement in its nutritional value is expected to increase consumer demands regarding health, nutrition and convenience. Several crops with nutraceutical value are being commercially utilized in several countries across the world, e.g. quality protein maize with high lysine and tryptophan content [1], purple colored carrots [2], potatoes, sweet potatoes and maize [3] with high anthocyanin content.

Anthocyanins have evoked the interest of number of researchers. Anthocyanins are normal constituents in colored fruits and vegetables. These can act as antioxidants and help in prevention of cardiovascular diseases [4], diabetes [5], inflammation, cancer [6], obesity [7] and aging [8]. Common wheat cultivars across the world are white (amber) in color. The colored wheat, rich in anthocyanin is quite uncommon. Purple color is localized to the pericarp [9, 10] whereas blue color to the aleurone [9, 11]. Black wheat resulted by the combination of genes for both purple and blue colors. Colored wheat has attracted the attention of many breeders across the world but these lines exhibit low yield due to linkage drag [12]. We have developed colored wheat lines with satisfactory yield potential and regional adaptation [9]). In this manuscript we have characterized these lines, with respect to colored donor and recipient high yielding white wheat lines. Colored wheat lines were characterized for anthocyanin content and antioxidant activities. For validation of health claiming benefits of color wheat lines, its anti-inflammatory effect was studied using macrophage cell line model. For assessment of bread making quality, chemical composition of colored advanced lines including minor components (minerals and vitamins) and rheological parameters were evaluated.

## Materials and methods

Plant material included one white wheat (cv, PBW621), three colored donor wheat lines (purple, blue, black) and three high yielding colored advanced breeding lines (purple, blue, black) selected from back crossed filial generations (BC_1_F_8_) of cross between white and donor colored wheat lines. They were grown and advanced in the farms of National Agri-Food Biotechnology Institute, Mohali, Punjab, India (30°44’10” N Latitude at an elevation of 351m above sea level) in 2015–2016.

### Anthocyanin extraction

Crude anthocyanins were extracted by using acidified methanol in 85:15, v/v (Methanol: 1N HCl) according to Boeing et al., [13] with slight modifications. Briefly, extracts were kept at -20°C for 24 h for precipitation of heavier molecules, clear supernatant was collected and filtered through syringe filters (0.45μm pore size) for further phytochemicals and antioxidant analysis.

For cell line studies pH of the acidified extracts was neutralized. The extracts were dried under vacuum and re-suspended in MQ water. This procedure of drying and re-suspension was repeated for at least 2–3 times until neutral pH was achieved.

### Determination of Total Anthocyanins Content (TAC)

TAC was determined using spectrophotometric method [14]). The absorbance of samples was measured at 520 nm, against distilled water as the blank. The data were expressed as micrograms (μg) of Cyanidin 3-glucoside (Cy 3-glu) equivalents per gram of dry matter.

### Determination of Soluble Phenolic Content (SPC)

The Folin-Ciocalteu method was used to determine the soluble phenolic content in acidified methanol extracts as described by Singleton et al. [15]. SPC was expressed as μg of gallic acid equivalents per gram of dry matter.

### Antioxidant activity

Total antioxidant activity of color wheat anthocyanin extract in comparison to common wheat extract was measured by three assays; DPPH (2,2-diphenyl-1-picrylhydrazyl), ABTS (2,2- (Azinobis (3-ethybenzothiazoline-6-sulfonic acid) diammonium salt) and PCL (Photochemiluminescence).

#### DPPH assay

DPPH assay was performed according to Hu & Kitts [16]. Absorbance was measured at 517 nm. Methanol solutions of known trolox concentrations were used for calibration curve and the results were expressed as mmol trolox equivalents/kg of sample dry weight (ppm). The antioxidant activity was calculated in terms of percentage (%) inhibition of DPPH, compared to blank.

#### ABTS assays

ABTS assay was performed according to Hu & Kitts [16]. Absorbance was measured at 734 nm. Methanolic solutions of known trolox concentrations were used for calibration curve and the results were expressed as mmol trolox equivalents/kg of sample dry weight (ppm). The antioxidant activity was calculated in terms of percentage (%) inhibition of ABTS, compared to blank.

#### PCL Assay

Antioxidant capacity of extracts was determined using Photochem^®^ instrument (Analytik Jena, Leipzig, Germany), by using water soluble substances kit (ACW) as per the manufacturers protocol. The ascorbic acid (μg/mg) standard was used for the calibration curve. Data acquisition and analysis was done through PCLsoft^®^ control and analysis software. Antioxidant activity was calculated as:

Concentration(μg/mg)=Q×Dilution×M×V/PV×Wt

Where, Q is nmol (ascorbic acid), M is molar mass of ascorbic acid i.e. 176.13 ng/ml, V is extraction volume in ml, Dilution is 1:10 dilution factor, PV is pipette volume in vial (μl), Wt is initial sample weight (mg).

Antioxidant capacity in terms of inhibition was measured according to lag_p_-lag_o_ from curves between luminescence on y-axis and time in seconds on x-axis. Lag is the interception point of the tangent of the curve with the x coordinate. Where, p is for sample and o is for blank.

### Ultra Performance Liquid Chromatography (UPLC) based separation of anthocyanins

Liquid chromatography of anthocyanin extracts was performed using a Waters Acquity Ultra-Performance^™^ LC system (Waters), equipped with a quaternary pump system. An Acquity BEH C-18 (50 mm×2.1 mm id, 1.7 μm particle size) column from Waters was used. Mobile phase consists of 1% aqueous formic acid (eluent A) and HPLC grade acetonitrile (eluent B) and gradient runs were within 6 minute which comprises 0–1 min, 95–5% B; 1–5 min, 80–20% B; 5.1–6 min, 95–5% B. The injection volume of extract applied to column was 5μl with flow rate of 4μl/min and the detection wavelengths were set at 520 nm. Anthocyanin standards included pelargonidin 3-glucoside (Pel 3-glc), cyanidin 3-glucoside (Cy 3-glc), cyanidin 3-rutinoside (Cy 3-rut), peonidin 3-galactoside (Pn 3-glc) and cyanidin chloride (Cy-Cl). The anthocyanin standard included in this study correspond to those reported in the literature for blue, purple, black and red cereals, except for cyanidin chloride, a pigment found in many red berries. We expected that cyanidin chloride should be present in colored wheat.

### Anthocyanins as anti-inflammatory agents

#### Cell culture

To study *in vitro* anti-inflammatory response of colored wheat lines, murine macrophage cell line RAW 264.7 was used. The culture medium was prepared using Dulbecco’s modified eagle medium (DMEM) media with 10% inactivated fetal bovine serum, 100 units/ml penicillin and 100 mg/ml streptomycin.

#### Cell viability test using MTT assay

Effect of anthocyanin rich wheat extract on cell viability was evaluated using MTT (3-(4,5-dimethylthiazol-2-yl)-2,5-diphenyltetrazolium bromide) assay kit. RAW 264.7 cells at exponentially growing phase were used. Cells were seeded into 96 well plates at density of 2 x 10^4^ cells/well plates/300 μl. Cells were challenged with 200, 400 and 800 μg/ml of crude anthocyanin extract of black and white wheat along with 20, 40 and 80 μl of water diluted media in the absence or presence of lipopolysaccharides (LPS) (1 μg/ml) for 24 h at 37°C in 5% CO_2_ incubator. After 24 hours, culture media were replaced with fresh medium containing 10μl of 12mM MTT solution. Incubation was continued and after 4 h, 100μl of SDS-HCl solution was added to dissolve MTT crystal and stop the MTT reaction. Absorbance was measured at 570 nm after 4 h of incubation. This experiment was performed in 6 replicates and repeated three times.

#### Protection against the oxidative damage-nitrite estimation

RAW264.7 macrophage cells were seeded into 48 well plates at density of 2.5 x 10^4^ cell/well/500μl media and then cultured for 24 h. Spent medium was replaced with a fresh one. At the same time cells were challenged with different concentrations of wheat extracts in the presence or absence of 1μg/ml LPS. After 24 h, NO level in the spent media was determined by mixing it with equal volume of Griess reagent and measuring the absorbance at 540 nm. A calibration curve of NO was constructed using sodium nitrite as a standard. Inhibition of nitric oxide level was expressed in terms of percentage (%) in comparison to control. This experiment was performed with at least 6 replicates and repeated three times.

#### Modulation of pro-inflammatory markers

The RayBio^®^ Mouse *in vitro* enzyme-linked immunosorbent assay (ELISA) kits were used for the quantitative measurement of pro-inflammatory cytokines, tumor necrosis factor-alpha (TNFα) and interleukin 1 beta (IL1β) in both basal condition and after cell treatment with LPS. ELISA experiment was performed as per manufacturer instructions.

### Nutritional and qualitative profiling

Moisture, carbohydrates, sugars, protein, dietary fibre and ash contents were determined according to the methods of association of analytical communities AOAC [17]. Gluten content and alveograph test were performed according to American association of cereal chemists (AACC) methods [18] and Sodium dodecyl sulphate sedimentation (SDSS) was determined by Garg et al. [19]. Vitamins (B3, B5) were measured by HPLC and minerals (Iron, zinc, copper, Manganese) were determined by ICP-MS according to AOAC [17]). Phytic acid and free phosphates were determined by Megazyme kit (K-PHYT 08/14) according to manufacturer’s instructions.

### Statistical analysis

Significance level for all biochemical assays and in vitro tests were evaluated by using one-way ANOVA followed by Waller Duncan or Tukey’s test using SPSS 17.0. Principal component analysis (PCA) was carried out by using XLSTAT 2016 software for the analysis of phytochemicals, antioxidant activity and nutritional components.

## Results

### Anthocyanin and Phenolic content of wheat lines

The TACs in the examined samples ranged from 13 ppm to 135 ppm. TAC followed the order of white<purple<blue<black wheat. White wheat exhibited very low TAC (Table 1). All colored lines had significantly higher TAC than white wheat. Significant variation in TAC was observed within the purple color lines. Our advanced purple line showed triple TAC than purple donor line (Table 1). Black donor and black advanced lines showed no significant differences in TAC. On the other hand, a significant variation was observed between blue donor and blue advanced lines.

**Table 1:** Phytochemicals content and antioxidant activity of colored wheat lines relative to white wheat.

Wheat lines	Phytochemicals		Antioxidant activity					
	TAC ppm	SPC ppm	DPPH		ABTS		PCL	
			%IN	TROx ppm	%IN	TROxppm	%IN	Asc ppm
White	13.0^**a**^±0.2	955.4^**a**^±7.4	69.4^**a**^±0.5	9.4^**a**^±0.3	6.4^**a**^±0.3	58.8^**a**^±0.5	48.3^**a**^±0.5	139^**a**^±3
Blue Donor	96.4^**c**^±3.1	1159.6^**b**^±90.2	74.8^**b**^±1.3	13.3^**b**^±0.8	26.1^**d**^±1.5	93.6^**d**^±2.7	97.2^**b**^±5.0	487^**c**^±30
Blue Advanced	120.6^**d**^±2.1	1202.7^**b**^±98.6	77.0^**c**^±0.6	14.2^**bc**^±0.4	28.2^**d**^±1.7	97.5^**d**^±2.9	161^**e**^±7.5	775^**e**^±108
Purple Donor	43.9^**b**^±0.7	1223^**b**^±75.6	77.0^**c**^±0.4	14.3^**bc**^±0.3	15.7^**b**^±0.6	75.2^**b**^±1.1	78.6^**b**^±0.9	325^**b**^±5
Purple Advanced	122.5^**d**^±9.8	1101.6^**b**^±42.4	77.8^**c**^±0.5	14.8^**c**^±0.3	17.3^**b**^±2.2	78.1^**b**^±3.9	120^**d**^±15.7	572^**d**^±92
Black Donor	135.8^**e**^±2.7	1174^**b**^±32.5	78.0^**c**^±0.5	15.1^**cd**^±0.6	22.3^**c**^±0.4	86.9^**c**^±0.8	181.8^**f**^±11.4	930^**f**^±65
Black Advanced	134^**e**^±8.4	1186.**7**^**b**^±81.8	79.8^**d**^±1.3	16.0^**d**^±0.8	22.2^**c**^±4.0	86.7^**c**^±7.0	146.7^e^±4.9	728^**e**^±28

TAC; Total anthocyanin content, SPC; Soluble phenolic content, DPPH; 2,2-Diphenyl-1-picryhydrazyl, ABTS; 2,2’-Azinobis(3-ethylbenzthiazoline)-6-sulfonic acid, PCL; Photo chemiluminescence, %IN; percentage inhibition, TROx; Trolox equivalent and Asc; Ascorbic acid equivalent.

Values followed by same letter in column are not significantly different (Tukey’s test or Duncan’s test, p>0.05)

The SPCs in the examined samples ranged from 955 ppm to 1223 ppm. The phenolic content of white wheat was significant (78% of highest value in colored wheat samples), compared to anthocyanin content (9.6% of highest value in colored wheat samples). All colored lines had significantly higher SPC than white wheat. Contrary to TAC results, no significant variation in SPC was observed within different colored wheat lines.

### Antioxidant activity of wheat lines

Three different methods were used to study the antioxidant potential of colored wheat lines, as different methods used can give varying results depending on the specific free radical being used as a reactant (Table 1).

#### DPPH assay

The % inhibition of DPPH assay varied from 69–80% and in terms of Trolox equivalent concentration from 9 ppm to 16 ppm with better separation among the studied wheat lines (Table 1). The antioxidant activity of colored wheat lines was significantly higher than white wheat. Antioxidant activity increased in the order of white<blue<purple<black wheat.

#### ABTS assay

The antioxidant activity was measured by ABTS assay as % inhibition that varied from 6–28% and as trolox equivalent concentration with variation from 59 to 98 ppm (Table 1). The antioxidant activity of colored wheat lines was significantly higher than white wheat. It followed a different trend than TAC as well as SPC i.e. white<black<purple<blue wheat. Insignificant variation in antioxidant activity was observed within purple, blue and black wheat lines.

#### PCL assay

The antioxidant capacity determined by PCL assay varied from 48–181% as % inhibition and from 139 to 930 in terms of concentration (ppm) (Table 1). The antioxidant activity of colored wheat lines was significantly higher than white wheat. It followed same trend as TAC, white<purple<blue<black wheat. Significant variation in antioxidant activity was observed within purple, blue and black wheat lines.

### PCA analysis of phytochemicals and antioxidant activity

**Fig 1:**
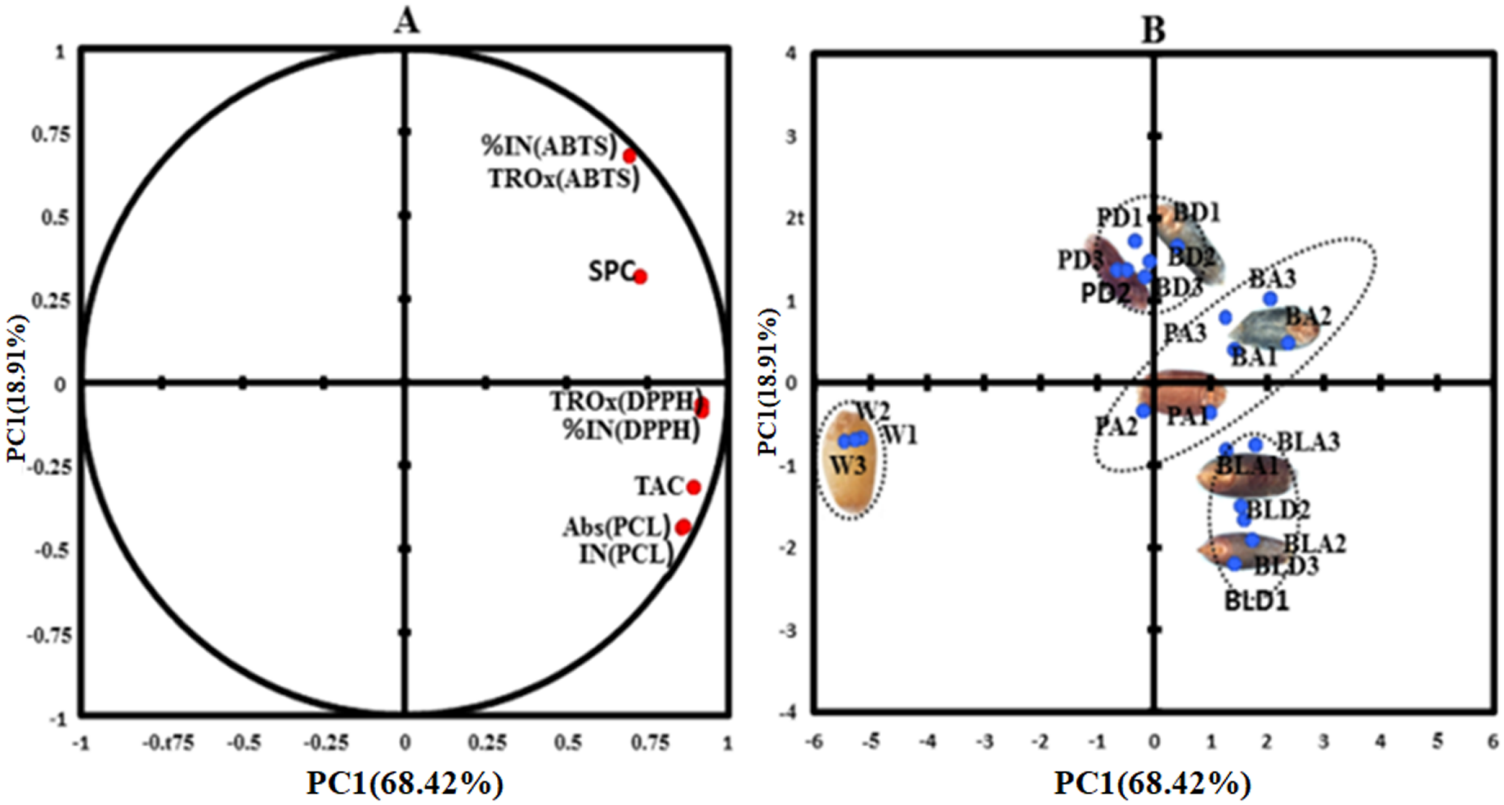
Principal component analysis (PCA) plots for phytochemicals and antioxidant activity. **(A)** Projection of variables (B) PCA score plots for wheat lines W-white wheat, BD- Blue donor, BA-blue advanced wheat, PD-purple donor wheat, PA-purple advanced wheat, BLD-black donor wheat, BLA-black advanced wheat.

PCA was carried out to find multiple correlations between phytochemicals and their antioxidant activities (Fig 1). PCA was separated into two main principal components (PC1 and PC2) and among them PC1 explained the 68.4% of the overall variation, whereas PC2 explained only 18.9%. This specified that PC1 itself explained maximum variability by all the variables (Fig 1). Loading factors also indicated high positive contributions (> 0.7) from all the variables (S1 Table) on PC1. A high positive correlation was observed between TAC-PCL assay (0.9) and SPC-DPPH assay (0.65) (S2 Table). ABTS assay had also shown positive correlation with TAC and SPC but correlation coefficient was less. TAC was closer to PCL assay based antioxidant activity. SPC lied between DPPH assay and ABTS assay being closer to DPPH assay (Fig 1A).

The sample score plot located the white wheat line on the left half of the plot, while all colored wheat lines were situated on the right-hand side of the figure (Fig 1B). Purple and blue donor lines grouped together. PC2 separated these lines from other white and colored lines. Black donor and black advanced lines formed a different cluster at the lower side of figure. Purple advanced and blue advanced lines formed fourth cluster.

### Chromatography configuration by UPLC

**Fig 2:**
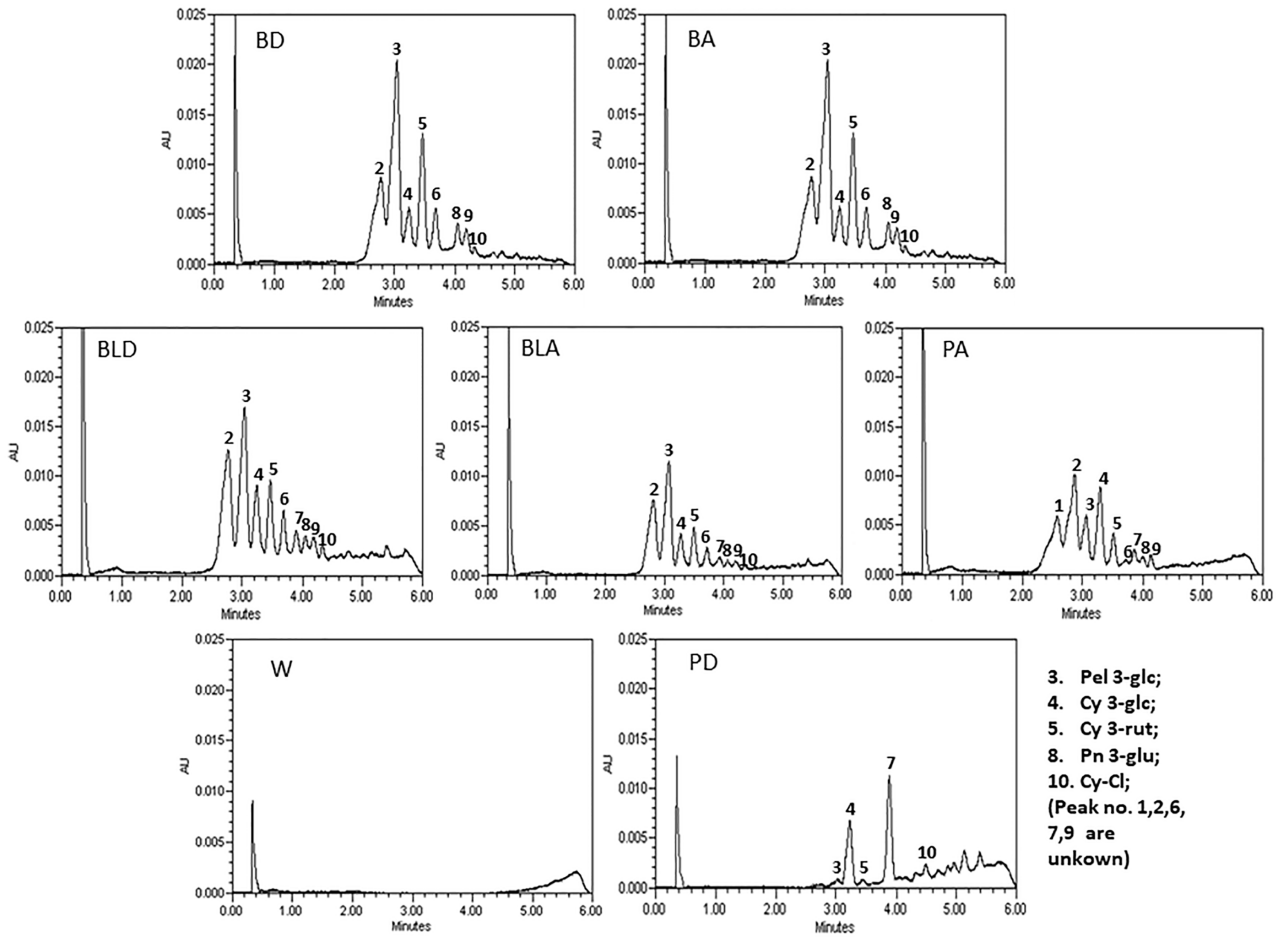
Chromatograms of anthocyanins in different colored wheat line extracts along with white wheat by UPLC. Peak 3: Pel 3-glc; 4: Cy 3-glc; 5: Cy 3-rut; 8: Pn 3-glc; 10: Cy-Cl and Peak No. 1, 2, 6, 7, 9 are unknown W-white wheat, BD- Blue donor, BA-blue advanced wheat, PD-purple donor wheat, PA-purple advanced wheat, BLD-black donor wheat, BLA-black advanced wheat.

Color composition of wheat donor and their respective advanced lines was studied by ultra-performance liquid chromatography (Fig 2). UPLC chromatograms of blue, purple and black wheat donor had shown characteristic peak patterns.

The chromatogram of donor black wheat line showed nine peaks. The identified peaks in black wheat donor were pelargonidin 3-glucoside (peak 3; Pel 3-glc), cyanidin 3-glucoside (peak 4; Cy 3-glc), cyanidin 3-rutinoside (peak 5; Cy 3-rut), peonidin 3-galactoside (peak 8; Pn 3-glc) and cyanidin chloride (peak 10; Cy-Cl). Similar chromatogram was observed in the selected black advanced line. The chromatogram of donor blue wheat showed eight peaks. The identified peaks were pelargonidin 3-glucoside (peak 3; Pel 3-glc), cyanidin 3-glucoside (peak 4; Cy 3-glc), cyanidin 3-rutinoside (peak 5; Cy 3-rut), peonidin 3-galactoside (peak 8; Pn 3-glc) and cyanidin chloride (peak 10; Cy-Cl). Similar chromatogram was observed in the selected blue advanced line. The chromatogram of donor purple wheat line showed five peaks. The identified peaks were peak pelargonidin 3-glucoside (peak 3; Pel 3-glc), cyanidin 3-glucoside (peak 4; Cy 3-glc), cyanidin 3-rutinoside (peak 5; Cy 3-rut) and cyanidin chloride (peak 10; Cy-Cl). Interestingly, the chromatogram of selected purple advanced line was quite different from donor purple wheat line. It showed nine peaks with different pattern of anthocyanin accumulation than donor purple wheat. The identified peaks were peak, cyanidin 3-glucoside (peak 4; Cy 3-glc), cyanidin 3-rutinoside (peak 5; Cy 3-rut) and peonidin 3-galactoside (peak 8; Pn 3-glc) While chromatogram patterns of black and blue wheat lines were quite similar, pattern of purple wheat chromatogram was significantly different. The peak pelargonidin 3-glucoside (peak 3; Pel 3-glc) with highest area under the curve in blue and black wheat showed much lower area under the curve in purple wheat.

### Anthocyanins as anti-inflammatory agents

#### Effect of wheat extracts on RAW 264.7 cell viability

**Fig 3:**
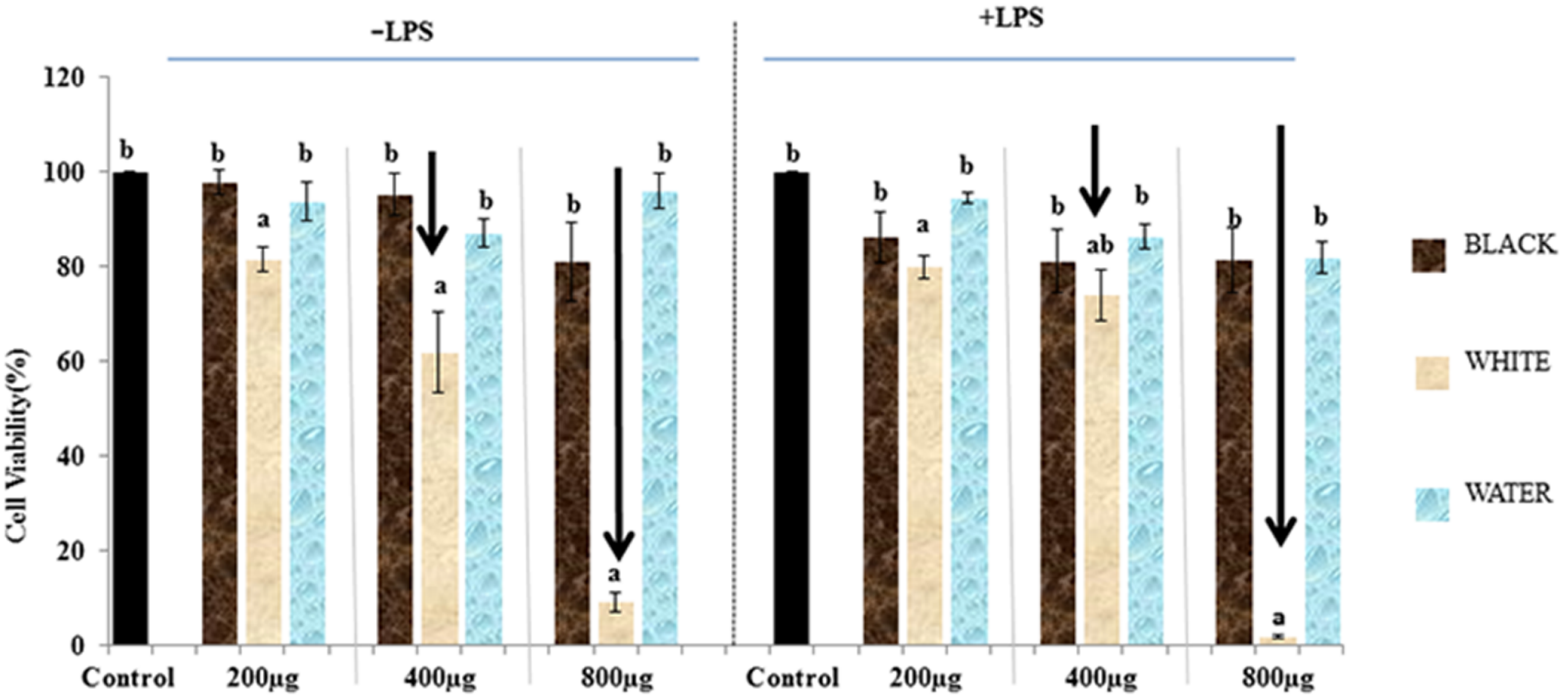
Cell viability evaluation by using MTT assay. Different letters signify significant differences (p< 0.05).

For cell viability evaluation, two lines with highest (black advanced) and lowest (white) anthocyanin content were selected. MTT assay indicated a dose dependent reduction in cell viability with white wheat extract (Fig 3) (p>0.05).

In the absence of LPS, there was decrease in viability with increase in doses of white wheat extract (200–800 μg/ml), with highest reduction of 80–90% at 800 μg/ml as compared to untreated control. A similar trend of cell viability was observed in the presence of LPS. In case of black wheat anthocyanin extract, at all the doses tested, cells exhibited >80% viability (Fig 3). This trend was also similar in presence of LPS. Using water as solvent had no adverse effect on cell viability. As at higher doses, white wheat had cyto-toxic effect, subsequently cell viability evaluation was carried out at lower doses (150–400 μg/ml) and that did not show significant effect on cell viability with or without LPS. Based on this experiment, 300 μg/ml dose was chosen for further evaluation of *in-vitro* anti-inflammatory activity.

#### Effect of wheat extracts on NO production in LPS-induced pro-inflammatory stress in macrophage cells

**Fig 4:**
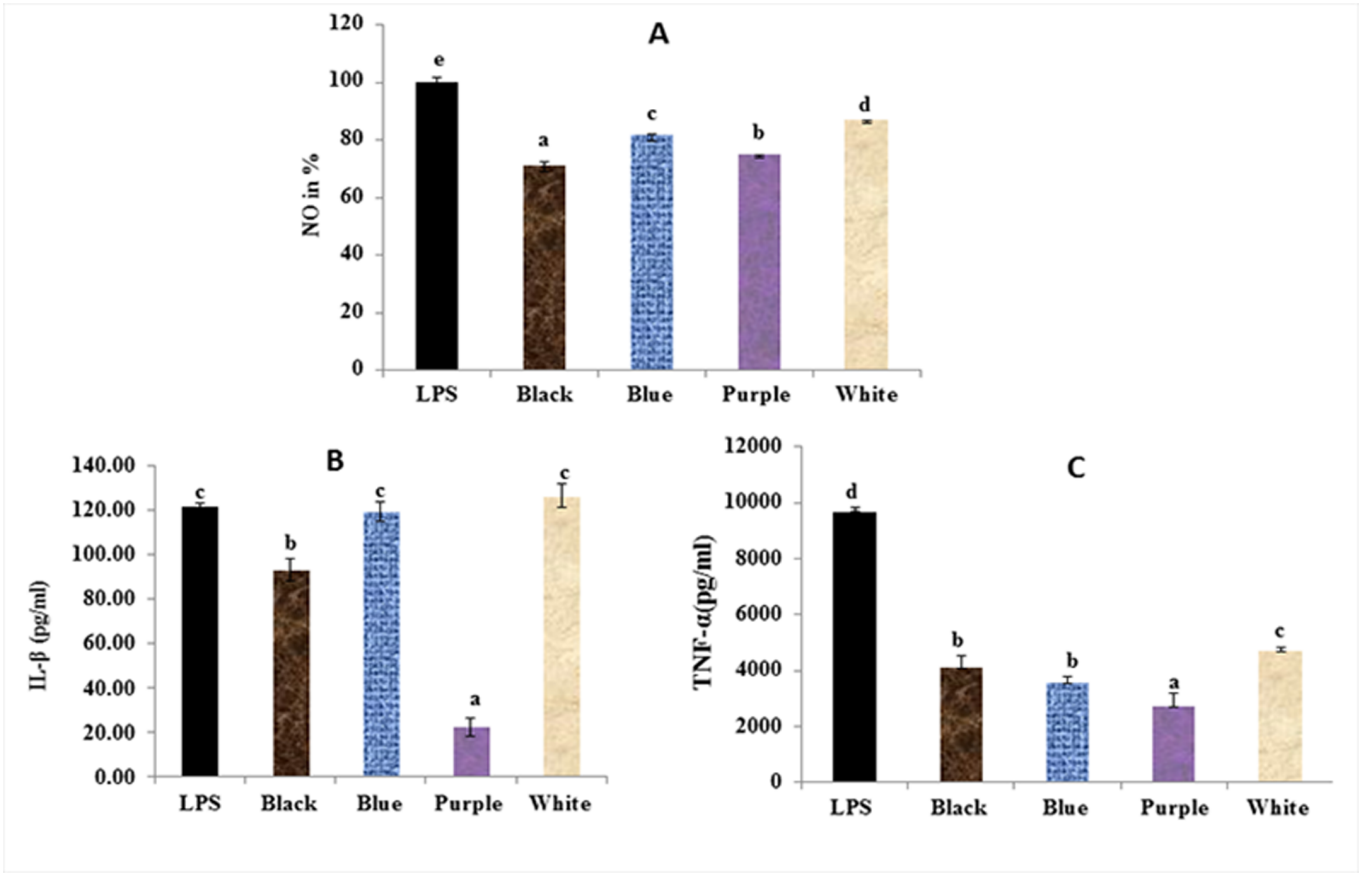
Estimation of anti-inflammatory effect of colored wheat lines on (A) NO level (B) LPS-induced pro-inflammatory cytokine IL-1β and (C) pro-inflammatory cytokine TNF-α level. Data was expressed as % of control and represents mean ± SD of 6 replicates. Same letters depict they are not significantly different (p<0.05).

Protective efficiency of colored wheat lines against LPS-induced pro-inflammatory stress was evaluated by estimating the release of NO by LPS treated RAW 264.7 cells (Fig 4A). Only advanced colored and white wheat lines were selected for NO estimation. At 300μg/ml concentration white and colored wheat lines showed reduction in NO level (Fig 4A) with respect to cells treated with LPS alone. Higher NO reduction was observed for colored wheat lines as compared to white wheat lines. Highest inhibition of NO production was observed in black wheat line, which was followed by purple, blue and then white wheat line. PCA assay indicated high positive correlation between reduction in NO levels and TAC (0.9), relative to SPC (0.3) (S3 Table).

#### Inhibitory effect of wheat extracts on pro-inflammatory cytokines in LPS-Induced RAW 264.7 Cell

Wheat extracts from colored wheat lines had inhibitory effects on the production of pro-inflammatory cytokines (TNF-α and IL-1β). LPS-induced production of IL-1β was significantly inhibited by most colored wheat extracts (Fig 4B). No significant difference in IL-1β was observed with white and blue wheat extracts. Purple wheat extract showed highest inhibition of IL-1β production, followed by black wheat extract. Different trend was observed in case of LPS-induced production of TNF-α (Fig 4C). Its production was inhibited by both colored and white wheat extracts, with colored wheat lines exhibiting higher inhibition than white wheat lines. Among the colored wheat lines, highest inhibition of TNF-α production was observed in case of purple wheat lines, followed by blue and then black wheat extracts. PCA assay indicated positive correlation within pro-inflammatory cytokines (TNF-α and IL-β) (0.7) and NO levels with IL- β (0.6) (S3 Table). Their correlation with TAC was positive (0.4) and with SPC was negative (-0.3).

### Nutritional and qualitative profiling

**Fig 5:**
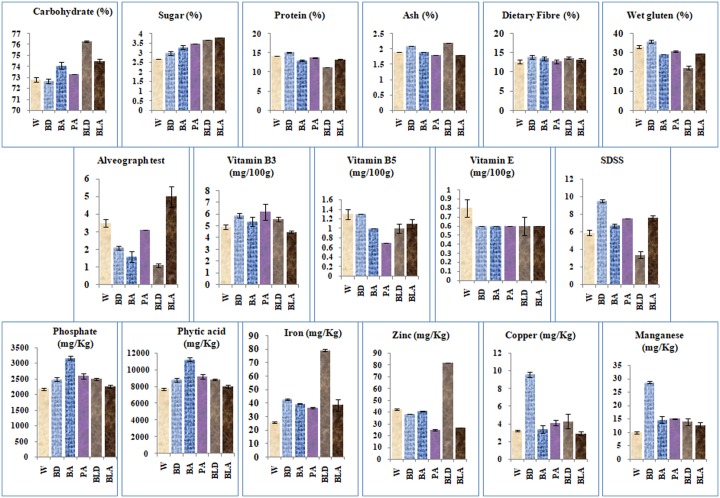
Nutritional components of color and white wheat lines. W-white wheat, BD- Blue donor, BA-blue advanced wheat, PA-purple advanced wheat, BLD-black donor wheat, BLA-black advanced wheat.

Nutritional profiling of colored wheat lines in comparison to white wheat lines was carried out in terms of major constituents (carbohydrates, sugar, protein, ash, dietary fibre), minor constituents (vitamins, essential metals and metal binding compound) and processing parameters in relation to end-use quality (Fig 5). Purple donor line was omitted from this study due to poor grain setting and seed availability. There were significant differences in the major constituents among the tested lines. Ash and dietary fibre content were higher in donor (black and blue) wheat lines compared to white and advanced colored lines. Protein content of advanced lines was in range of white wheat line, being higher in blue donor and lower in black donor line. Carbohydrates and sugar content followed the same trend, being low in blue donor and white wheat lines and high in black donor and advanced lines. Significant differences were also observed in amount of minor constituents among the tested lines. Vitamin B3 was higher in blue donor, B5 in blue donor and white wheat. Iron and zinc contents were higher in black donor and copper and manganese contents were higher in blue donor line. Except for zinc all other metals were lowest in white wheat. In case of white wheat phosphate and phytic acid content were lowest. Highest phosphate and phytic acid content was observed in blue advanced line. Among the processing quality parameters, SDS sedimentation value and gluten content followed the same trend as protein content, being highest in blue donor and lowest in black donor line with all advanced colored lines in the range of white wheat line. For alveograph, lowest value was observed in black donor line with lowest protein content, SDS sedimentation value and gluten content. Highest value was observed in black advanced line followed by white and purple advanced lines.

**Fig 6:**
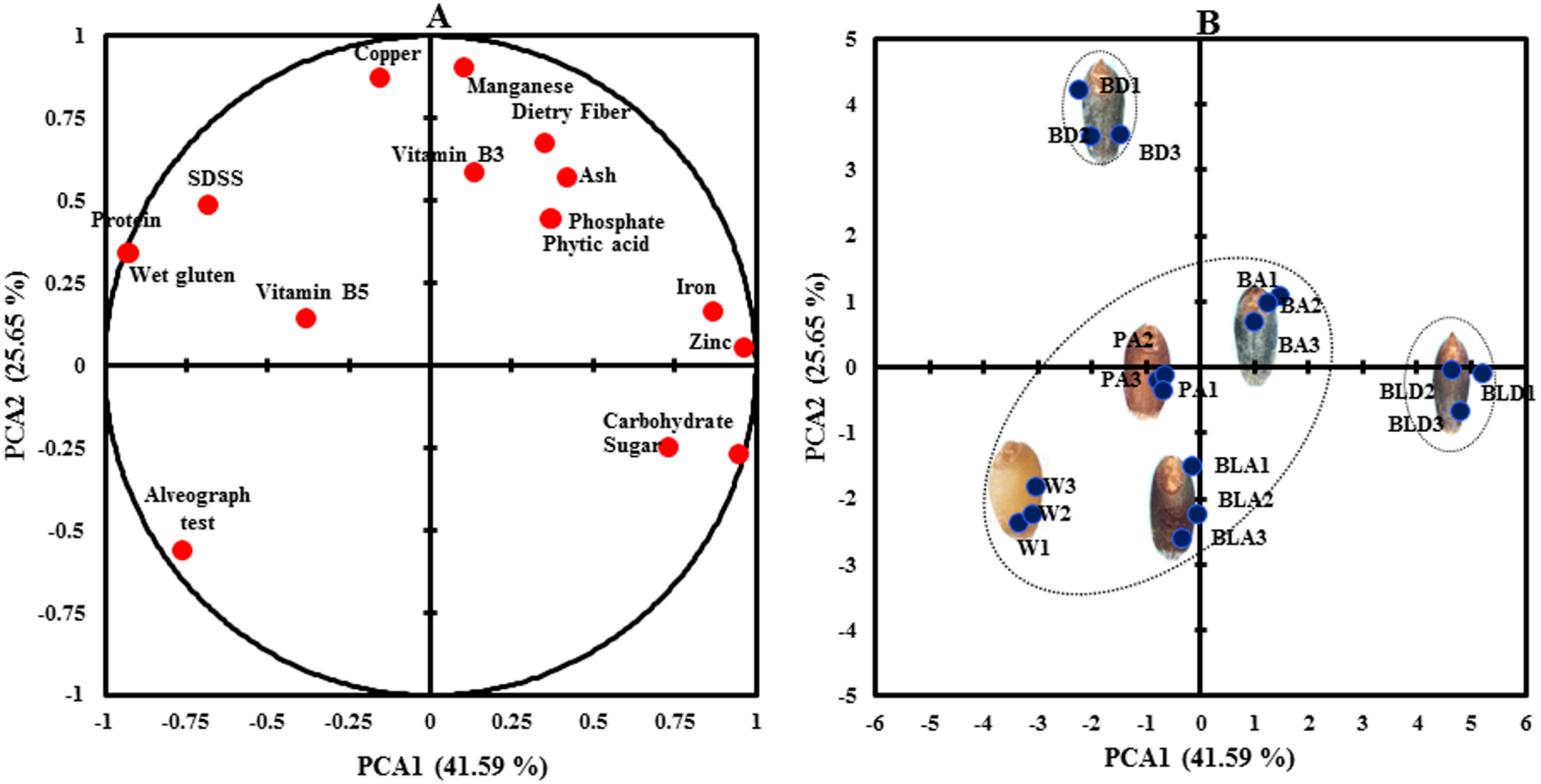
Principal component analysis based on nutritional components. (A) Projection of 17 variables tested on the plane formed by PC1 and PC2 (B) The scatter plot reports the projection of 6 wheat lines. Advanced wheat lines grouped with white wheat line.

Multiple inter-correlations between wheat lines and analyzed nutritional parameters were determined by PCA (Fig 6). PC1 accounted for the 41.59% of the total variance and showed high positive loadings (>0.55) for protein, wet gluten, alveograph test and SDSS (S4 Table). This result indicated a significant multiple correlation among the mentioned variables and they all account for the wheat endosperm components. The PC2 explained 25.65% of the total variance and showed high positive loadings (>0.55) for ash, dietary fibre, vitamin B3, copper and manganese. All these variables account for the wheat bran components. Projection of wheat lines in 2-dimentional spaces of the PC1 and PC2 loading factors could be differentiated into three main clusters (Fig 6). Blue donor and black donor lines formed two separate clusters. White wheat and all advanced lines formed third cluster. With respect to second PCA white wheat expressed the lowest bran components. On considering the placement of each wheat variety with respect to PC2, the relative ranking for bran layer components was white wheat<black advanced<purple advanced<blue advanced<blue donor<black donor. Thus purple advance line was showing almost equal contribution towards bran and endosperm components.

## Discussion

Anthocyanin rich colored wheat is drawing attention of scientists and food industry across the world because of its potential as vibrant food colorant, nutraceutical ingredient and functional food [20, 21]. Anthocyanins are water soluble pigments that belong to class flavonoids [22]. Later is part of polyphenols and include anthocyanidins, flavonols, flavones, isoflavones, flavanones and flavanols. Polyphenols include phenolic acids, flavonoids, stilbenes and lignans. The most common white wheat grains are rich source of phenolic acids most abundant being ferulic acid that acts as a potent antioxidant [22]. Therefore antioxidant activity of white wheat is majorly associated with its phenolic acids and in colored wheat, additional effect is due to high anthocyanin content. Both phenolic and anthocyanin contents were estimated in the current study. Phenolic content of colored wheat lines was significantly higher than white wheat line, but there were no significant differences in the phenolic content among different colored wheat lines under study. On the other hand, there was a definite trend in the anthocyanin content, among different colored wheat lines black>blue>purple>white. This indicated that anthocyanin and phenolic content are unrelated and we can estimate antioxidant activity contributed by anthocyanins.

Antioxidant activity includes scavenging of reactive oxygen species (ROS—O_2_^•−^, H_2_O_2_, RO·, ROO·, OH·, HOCl·), reactive nitrogen species (RNS—NO·, ONOO^−^), reducing capacity; metal chelating capacity; activity as anti-oxidative enzyme; inhibition of oxidative enzymes [23]. There are different methods for estimation of scavenging of each type of free radical by different antioxidants. We used four different methods for antioxidant activity estimation: DPPH, ABTS, PCL and NO assays. Highest positive correlation was observed between anthocyanin content and PCL assay. The correlation of TAC with other methods was also positive but relatively lower. For SPC highest positive correlation was observed with DPPH assay but it was not to the level of TAC—PCL assay. Ours results indicated that antioxidant activity of colored wheat is associated with its anthocyanin content in first place and increased phenolic content in second place. There had been some disagreement about the relationship between phenolic content, anthocyanin content and antioxidant activity [24, 25]. There are different methods for estimation of scavenging of each type of free radical and it can be misinterpreted depending upon the method used. In DPPH assay (DPPH·+RH = DPPHH+R·), nitrogen centered stable DPPH free radical is reduced by receiving hydrogen atom from hydrophilic and lipophilic antioxidants [26]. In ABTS assay (ABTS^+^·+e^-^ = ABTS, ABTS^+^·+RH = ABTS^+^ +R·), nitrogen centered cation ABTS free radicle is reduced by receiving electron and hydrogen atom from both hydrophilic and lipophilic antioxidants. Both DPPH and ABTS assays do not measure antioxidant activity against biologically relevant radicles. Both methods are easy and extensively used for antioxidant assays. But our study indicated poor correlation between these assays and anthocyanin content. Their correlation with phenolic content was relatively better.

In PCL assay (L^-^·+O_2_ = LO^2-^) carbon centered luminol radical reacts with oxygen to form biologically relevant peroxyl radical, production of later is prevented by hydrophilic antioxidants. Anthocyanins are hydrophilic polyphenols and highly soluble in water. Aqueous solubility of different phenolic acids is different and most abundant phenolic acid in wheat i.e. ferulic acid has low solubility in water [27]. Therefore, PCL assay was predicted as best method for anthocyanin based antioxidant activity estimation in our study.

Characterization of anthocyanins by using liquid chromatography is an authenticate method and the observed peak patterns of different color wheat lines provide us the insight of anthocyanin composition. Previous studies have indicated that cyanidin 3-glucoside was a major anthocyanin in purple wheat [11, 12, 28, 29] while delphinidin 3-glucoside as principal component in blue wheat [23, 27]. There had been conflicting reports on major anthocyanin derivatives found in colored wheat. Some reports mention Cy 3-glu as major anthocyanin in purple wheat while others Cy 3-rut [30]. Our results indicated that Cy 3-rut had higher contribution in blue wheat and Cy 3-glu had higher contribution in purple wheat. While the chromatographic pattern of donor and advanced lines of blue and black wheat lines was similar to each other, purple advanced line showed significantly different pattern than purple donor wheat line. It was also different from black and blue wheat lines. It indicates that background of plant plays significant role in expression of different anthocyanins. Quite different anthocyanins can express depending upon the donor and recipient cultivars used for the development of germplasm. Therefore, anthocyanin accumulation and thus chromatographic pattern may differ between different colored wheat lines being used by different research groups in different countries. In addition growing location and environmental conditions also affect anthocyanin accumulation [28].

LPS treatment in macrophages causes NO and pro-inflammatory cytokines production. All these are biologically relevant and are reduced by antioxidants. High positive correlation was observed between TAC and NO assay, although correlation between TAC and pro-inflammatory cytokines was relatively less. There was insignificant correlation between SPC and pro-inflammatory cytokines.

Thus both biologically relevant PCL assay and cell line based assay indicated higher positive correlation with TAC than biologically non-relevant DPPH and ABTS assays. Cell line based studies are difficult and time consuming. On the other hand PCL assay is quick and highly sensitive. Thus PCL assay was best method for determination of antioxidant potential of colored wheat in comparison to white wheat. In addition to antioxidant potential, colored wheat lines had positive effect on cell viability. At higher doses, white wheat lines showed a substantial reduction in cell viability that was not observed in colored wheat lines. This might be due to high anthocyanin content in these lines that are exerting positive effect on cell viability.

Although purple wheat line had low TAC and PCL assay based antioxidant activity respect to blue and black lines, they exhibited higher inhibitory effects on the production of pro-inflammatory cytokines in cell line based assays. This might be due to higher concentration of acylated anthocyanins in purple wheat [28] with better stability and bioavailability [29]. Acylated anthocyanins are very low in concentration relative to non-acylated ones and are often overlooked.

We have developed colored wheat lines adapted to local climatic conditions. It is important to understand their potential for product making. Anthocyanin content and antioxidant activity of the advanced wheat lines was either equal to or higher than donor lines. Especially in case of purple wheat lines with three time higher anthocyanin content than donor line.

Donor wheat lines were exotic lines with poor yield and agronomical traits compared to normal white wheat cultivars. Seeds were smaller with less flour extraction rate. As expected, donor wheat lines exhibited higher ash and dietary fibre content. Appropriate protein content is required for product-based utilization. Protein content of advanced lines was in range of recipient white line, indicating that developed colored lines have similar potential for product development as white wheat. The sugars, starch and dietary fibre constitute carbohydrates. Our advanced lines had satisfactory carbohydrate content. Higher sugar content in some colored wheat lines might pave the way for their better acceptability by the consumer. Vitamins and minerals are mainly located in the bran layers and germ [30]. Their higher content in donor lines was expected due to relatively poor yield, lower flour and starch content: so that the contribution of bran layers towards the total seed weight is higher. Phytic acid binds with essential metal ions and reduces their bioavailability [30]. Wheat lines with higher metal content are expected to have higher phytic acid content. Although this trend was observed in white wheat line, same was not true for colored lines, indicating that it will be possible to breed colored wheat lines with high yield, high metal ion content and low phytic acid content to add to their bioavailability. Blue and black colored wheat lines had better micronutrients than white wheat as reported previously [31]. It might be due to blue aleurone layer in these lines. Purple color is in the pericarp, and not in the aleurone. Aleurone layer is the only living part in wheat seed and is reservoir of micronutrients in wheat. Higher anthocyanin content and thus antioxidant content in aleurone layer might be associated with their better development and micronutrient accumulation. Iron and zinc are important micronutrients and their deficiency is prevalent in India. Several research groups are involved in improvement of iron and zinc content of wheat. Our colored wheat lines with high anthocyanin content also have high iron and zinc content and thus are double biofortified lines that are expected to have significant effect on human health. Further these lines possess all the features required for commercial product development.

## Conclusion

Biofortified crops have huge market potential. Advanced high yielding black, blue and purple wheat lines investigated in the manuscript showed high anthocyanin content, antioxidant and anti-inflammatory activities. Apart from having health promoting effects, these lines possessed all the features required for commercial product development, paving way for their industrial utilization.

Measurement of antioxidant activity is a routine experiment and DPPH assay is most convenient, cheap and most commonly used method. But we found that PCL assay as best method for anthocyanin based antioxidant activity measurement.

## Supporting information

S1 Table Factor loadings of the 8 variables used in the principal component analysis of phytochemicals and their antioxidant activity. (PDF)

S2 Table Correlation coefficients between phytochemicals and their antioxidant activities. (PDF)

S3 Table Correlation coefficients between phytochemicals and *in vitro* cell line assays. (PDF)

S4 Table Factor loadings of the 17 variables used in the principal component analysis of nutritional components. (PDF)
