# Supplementary material for: Anthocyanin bio-fortified colored wheat: Nutritional and functional characterization
Source: PLoS One. 2018 Apr 4;13(4):e0194367. doi: 10.1371/journal.pone.0194367 (PMC5884506; doi:10.1371/journal.pone.0194367)
Supplement: S1 Table — (PDF) [file pone.0194367.s001.pdf]

S1 Table

	PC1	PC2
**TAC**	**0.896**	-0.316
**SPC**	**0.730**	0.314
**%IN(DPPH)**	**0.919**	-0.067
**TROx(DPPH)**	**0.920**	-0.088
**%IN(ABTS)**	**0.696**	**0.679**
**TROx(ABTS)**	**0.696**	**0.679**
**IN(PCL)**	**0.857**	-0.439
**Abs(PCL)**	**0.863**	-0.432
