# Supplementary material for: Anthocyanin bio-fortified colored wheat: Nutritional and functional characterization
Source: PLoS One. 2018 Apr 4;13(4):e0194367. doi: 10.1371/journal.pone.0194367 (PMC5884506; doi:10.1371/journal.pone.0194367)
Supplement: S2 Table — (PDF) [file pone.0194367.s002.pdf]

S2 Table

Variables	TAC	SPC	%IN (DPPH)	TROx (DPPH)	%IN (ABTS)	TROx (ABTS)	IN (PCL)	Abs (PCL)
**TAC**	**1**	0.447	**0.804**	**0.808**	0.454	0.454	**0.911**	**0.915**
**SPC**	0.447	**1**	**0.647**	**0.646**	**0.607**	**0.607**	**0.483**	**0.487**
**%IN(DPPH)**	**0.804**	**0.647**	**1**	**0.998**	**0.541**	**0.541**	**0.722**	**0.728**
**TROx(DPPH)**	**0.808**	**0.646**	**0.998**	**1**	**0.527**	**0.527**	**0.734**	**0.740**
**%IN(ABTS)**	**0.454**	**0.607**	**0.541**	**0.527**	**1**	**1.000**	0.335	0.344
**TROx(ABTS)**	**0.454**	**0.607**	**0.541**	**0.527**	**1.000**	**1**	0.335	0.344
**IN(PCL)**	**0.911**	**0.483**	**0.722**	**0.734**	0.335	0.335	**1**	**1.000**
**Abs(PCL)**	**0.915**	**0.487**	**0.728**	**0.740**	0.344	0.344	**1.000**	**1**

Values in bold are different from 0 with a significance level alpha=0.05. Values in bold within the same factor indicate the variable with the largest correlation
