# Supplementary material for: Anthocyanin bio-fortified colored wheat: Nutritional and functional characterization
Source: PLoS One. 2018 Apr 4;13(4):e0194367. doi: 10.1371/journal.pone.0194367 (PMC5884506; doi:10.1371/journal.pone.0194367)
Supplement: S3 Table — (PDF) [file pone.0194367.s003.pdf]

S3 Table

Variables	TAC	SPC	NO (%)	IL6-α	TNF-á
**TAC**	**1**	0.328	**0.872**	0.400	0.429
**SPC**	0.328	**1**	0.313	-0.348	-0.181
**NO (%)**	**0.872**	0.313	**1**	**0.595**	0.322
**IL6-α**	0.400	-0.348	**0.595**	**1**	**0.678**
**TNF-α**	0.429	-0.181	0.322	**0.678**	**1**

Values in bold are different from 0 with a significance level alpha=0.05
