# Supplementary material for: Anthocyanin bio-fortified colored wheat: Nutritional and functional characterization
Source: PLoS One. 2018 Apr 4;13(4):e0194367. doi: 10.1371/journal.pone.0194367 (PMC5884506; doi:10.1371/journal.pone.0194367)
Supplement: S4 Table — (PDF) [file pone.0194367.s004.pdf]

S4 Table

	PC1	PC2
**Carbohydrate (%)**	**-0.934**	-0.227
**SUGAR(%)**	**-0.655**	-0.283
**Protein(%)**	**0.945**	0.315
**Ash(%)**	-0.441	**0.678**
**Dietry Fiber(%)**	-0.301	**0.670**
**wet gluten (%)**	**0.943**	0.310
**Alveograph test**	**0.597**	-0.639
**Vitamin B3 (mg/100g)**	-0.142	**0.577**
**Vitamin B5 (mg/100g)**	0.369	0.248
**vitamin E(mg/100g)**	**0.547**	-0.489
**SDSS**	**0.770**	0.420
**Phosphate (mg/Kg)**	-0.346	0.379
**Phytic acid (mg/Kg)**	-0.347	0.381
**Iron (mg/Kg)**	-0.900	0.233
**Zinc (mg/Kg)**	-0.800	0.154
**Copper (mg/Kg)**	0.245	**0.901**
**Manganese (mg/Kg)**	0.222	**0.918**
